# Feasibility Study of NMR Based Serum Metabolomic Profiling to Animal Health Monitoring: A Case Study on Iron Storage Disease in Captive Sumatran Rhinoceros (*Dicerorhinus sumatrensis*)

**DOI:** 10.1371/journal.pone.0156318

**Published:** 2016-05-27

**Authors:** Miki Watanabe, Terri L. Roth, Stuart J. Bauer, Adam Lane, Lindsey E. Romick-Rosendale

**Affiliations:** 1 Division of Pathology, Cincinnati Children’s Hospital Medical Center, Cincinnati, Ohio, United States of America; 2 Division of Experimental Hematology and Cancer Biology, Cancer and Blood Disease Institute, Cincinnati Children’s Hospital Medical Center, Cincinnati, Ohio, United States of America; 3 Division of Biostatistics and Epidemiology, Cincinnati Children’s Hospital Medical Center, Cincinnati, Ohio, United States of America; 4 Center for Conservation and Research of Endangered Wildlife, Cincinnati Zoo and Botanical Garden, Cincinnati, Ohio, United States of America; The Pennsylvania State University Hershey Medical Center, UNITED STATES

## Abstract

A variety of wildlife species maintained in captivity are susceptible to iron storage disease (ISD), or hemochromatosis, a disease resulting from the deposition of excess iron into insoluble iron clusters in soft tissue. Sumatran rhinoceros (*Dicerorhinus sumatrensis*) is one of the rhinoceros species that has evolutionarily adapted to a low-iron diet and is susceptible to iron overload. Hemosiderosis is reported at necropsy in many African black and Sumatran rhinoceroses but only a small number of animals reportedly die from hemochromatosis. The underlying cause and reasons for differences in susceptibility to hemochromatosis within the taxon remains unclear. Although serum ferritin concentrations have been useful in monitoring the progression of ISD in many species, there is some question regarding their value in diagnosing hemochromatosis in the Sumatran rhino. To investigate the metabolic changes during the development of hemochromatosis and possibly increase our understanding of its progression and individual susceptibility differences, the serum metabolome from a Sumatran rhinoceros was investigated by nuclear magnetic resonance (NMR)-based metabolomics. The study involved samples from female rhinoceros at the Cincinnati Zoo (n = 3), including two animals that died from liver failure caused by ISD, and the Sungai Dusun Rhinoceros Conservation Centre in Peninsular Malaysia (n = 4). Principal component analysis was performed to visually and statistically compare the metabolic profiles of the healthy animals. The results indicated that significant differences were present between the animals at the zoo and the animals in the conservation center. A comparison of the 43 serum metabolomes of three zoo rhinoceros showed two distinct groupings, healthy (n = 30) and unhealthy (n = 13). A total of eighteen altered metabolites were identified in healthy versus unhealthy samples. Results strongly suggest that NMR-based metabolomics is a valuable tool for animal health monitoring and may provide insight into the progression of this and other insidious diseases.

## Introduction

Iron storage disease (ISD), the deposition of excess body iron into insoluble iron clusters in soft tissue, is a health concern in many species in captivity [1, 2]. The Sumatran rhinoceros (*Dicerorhinus sumatrensis*) is one of two browsing rhinoceros species that have evolutionarily adapted to a low-iron diet and is hence susceptible to iron overload [3]. It has been speculated that the lack of browse diversity and/or environmental conditions such as low exercise level and absence of hemoparasites are associated with the elevated metrics of iron loading in captive Sumatran rhinoceroses, however, the underlying cause of disease and the differences in individual susceptibility to ISD remain unclear [3, 4].

In humans, ISD or hemochromatosis is often associated with an autosomal recessive disorder caused by a mutation on the hemochromatosis (HFE) gene, which leads to increased iron absorption [5]. The HFE gene found in four rhinoceros species has been sequenced and mutations have been identified in two of the browser rhinoceros species [6]. However, a more recent study has challenged the relevance of this mutation as the underlying cause for ISD in captive rhinoceros species [7].

Most currently available diagnostic tests for animal disease rely on techniques such as a dipstick test, enzyme-linked immunoabsorbant assay (ELISA), or polymerase chain reaction (PCR). These methods all require the prior identification of target molecules, genes or proteins, and the development/validation of the detection method *e*.*g*. a specific antibody or primer for a species specific biomarker. Serum ferritin concentrations and transferrin saturation have been the two biomarkers most commonly employed to monitor iron loads in the rhinoceros [4]. Historically, these tests relied on an ELISA developed with antibodies to horse ferritin, but recently, an ELISA has been developed for Sumatran rhinos using monoclonal antibodies generated against Sumatran rhinoceros ferritin [8]. Regardless, serum ferritin concentrations may not be diagnostic of this disease because ferritin is an acute phase inflammatory protein and is not specific to ISD. Furthermore, the most accurate measurements require direct analysis of hepatic tissue [9] which is not feasible in a rhinoceros. Increases in serum transferrin saturation and ferritin levels have been shown to correlate with time in captivity in black rhinoceroses, however, the same has not been demonstrated for Sumatran rhinoceroses. Preliminary evidence suggests disparity in serum ferritin concentrations among Sumatran rhinoceroses in native and non-native habitats [4, 9]. In addition, the lack of knowledge regarding what normal (wild type) ferritin concentrations are and the maximum, healthy ferritin concentrations that a rhinoceros can tolerate creates another challenge in disease diagnosis. For these reasons, there is an urgent need to find other biomarkers specific to this disease that can be used for the development of diagnostic tests that are reliable, reproducible and applicable to any non-model animal species on a global basis.

Metabolomics monitors the alterations in metabolite concentrations caused by stimuli in given biological samples and has been demonstrated as a useful tool in many areas such as medical, environmental, and basic biological research [10–14]. This approach provides untargeted and comprehensive profiles of small molecules in body fluids and/or tissue extracts. The comparison of metabolic signatures can be used to identify differences due to age, gender, disease development, drug treatments, diets and living environments. One of the advantages of using this approach is that neither a specific marker for each condition nor species specific databases are required. Metabolomics has the potential to be more suitable over currently available tools for health monitoring of non-model species such as protected or endangered animals. This potential has been demonstrated in several species such as fish [15, 16], crustaceans [17, 18], mussels [19–21], whale sharks [22], and black bears [23]. In addition, metabolomics analyses provide a large amount of information from samples that can be obtained by non-lethal and minimally invasive methods *e*.*g*. urine, feces, and blood.

The objectives of this study are to demonstrate the ability of NMR-based metabolomics to differentiate the health status of individual Sumatran rhinoceroses and to identify metabolites that are altered during disease onset and/or progression. In addition, the effect of the living environment on the serum metabolome is assessed in this study using samples collected from seven individual Sumatran rhinoceros from two locations, the Cincinnati Zoo and Botanical Garden in Cincinnati, Ohio and the Sumatran Rhinoceros Conservation Centre in Peninsular Malaysia.

## Materials and Methods

### Serum samples

The serum samples analyzed in this study were obtained opportunistically from a bank of samples previously collected from seven female Sumatran rhinoceros between years 1997 to 2014 (Table 1) as part of their regular health monitoring program. Because so few Sumatran rhinoceros currently exist, serial samples collected over time from the same individuals were used to observe the alteration in the serum metabolome during disease development. To avoid the potential influences that gender may have on the serum metabolome, only female rhinoceros were profiled for this study. One sample from each of four female Sumatran rhinoceroses (Rhino-4, Rhino-5, Rhino-6 and Rhino-7) maintained in captivity at the Sungai Dusun Sumatran Rhinoceros Conservation Centre (SRCC) in Peninsular Malaysia were analyzed in this study to determine if serum metabolomes of this species are affected by environmental differences such as habitats/diets. Multiple samples collected over several years from three rhinoceroses maintained at the Cincinnati Zoo, (Rhino-1, Rhino-2 and Rhino-3) were analyzed for differences between healthy and unhealthy states. Two of the animals, Rhino-1 and -2 died from hemochromatosis. The healthy sample group in this study included samples from Rhino-3, a healthy control animal (n = 5), as well as the samples from Rhino-1 (n = 14) and Rhino-2 (n = 11) collected 11 months and 2 years prior to any clinical signs of hemochromatosis, respectively. Rhino-1 (n = 10), and Rhino-2 (n = 3) samples collected after diagnosis were categorized as unhealthy.

**Table 1:** List of samples from Sumatran rhinoceroses used in this study.

			Sample collection year			
Sample	Gender	Living environment	Healthy	n	Sick	n
Rhino_1	F	Cincinnati zoo, OH	2000–2012	14	2013–2014	10
Rhino_2	F	Cincinnati zoo, OH	1997–2008	11	2009	3
Rhino_3	F	Cincinnati zoo, OH	1997–2000	5		
Rhino_4	F	The Sumatran Rhinoceros Conservation Centre, Malaysia	2003	1		
Rhino_5	F	The Sumatran Rhinoceros Conservation Centre, Malaysia	2003	1		
Rhino_6	F	The Sumatran Rhinoceros Conservation Centre, Malaysia	2003	1		
Rhino_7	F	The Sumatran Rhinoceros Conservation Centre, Malaysia	2003	1		

### Sample Collection

Female Sumatran rhinoceroses at the Cincinnati Zoo were trained through operant conditioning to allow blood collection from the veins that run along the back of the ears. Rhinoceroses at the SRCC in Malaysia were conditioned for blood collection from a lateral vein in the tail. No sedation or anesthesia was necessary, and animals were not fasted prior to blood collection. A tourniquet was placed at the base of the ear or base of tail, and a 23-gauge butterfly catheter was inserted into the vein. Blood from the ear vein was collected into a 6 mL syringe and emptied into a red top serum separator collection tube. Blood from the tail was collected directly in the vacutainer red top serum separator tube. After approximately 30–60 minutes, samples were centrifuged (1300 *xg*) and the recovered serum stored in ~1.0 mL aliquots at -80°C until they were thawed for analysis.

### Sample preparation

To obtain both polar and non-polar fractions of the serum for future analysis, all samples were extracted using a modified Bligh and Dyer extraction method. Methanol-chloroform-water extraction of the serum samples was performed as previously described [24]. Briefly, the appropriate volumes of solvents (final constant ratio of 2:2:1.8 of chloroform: methanol: water) were added to serum samples [25, 26]. The top layer, i.e. the hydrophilic extract, was dried in a vacuum centrifuge for approximately 2 hrs at room temperature and stored at -80°C until further preparation for NMR data collection. On the day of the data collection, dried polar extracts were re-hydrated with 600 μL of NMR buffer containing 100 mM phosphate buffer, pH 7.3, 1 mM TMSP (3-Trimethylsilyl 2,2,3,3-d4 propionate, CAS 24493-21-8), and 1 mg/mL NaN3 (sodium azide CAS 26627-22-8) prepared in D_2_O. A550 μL aliquot of each sample was then transferred into 5 mm NMR tubes (Norell).

### Quality control

To assess the reproducibility of the sample extraction, sample stability and analytical methods, quality control samples were prepared and analyzed along with the test samples. In-house prepared pooled human plasma control material (PCM) samples and a solvent blank sample were extracted with test samples in each batch. In total, five PCM samples and blank samples were extracted in this study.

### Data collection

One-dimensional ^1^H NMR spectra were recorded on all samples using the Carr-Purcell-Meiboon-Gill (CPMG) pulse sequence with presaturation of the water peak on a 600 MHz INOVA spectrometer. Experiments were run with 4 dummy scans (DS) and 256 acquisition scans (NS) with an acquisition time (AQ) of 2.09s, a relaxation delay (D1) of 4.0s and a mixing time was 60ms. The spectral width was 26 ppm, and 64K real data points were collected. Two-dimensional ^13^C edited heteronuclear single quantum correlation (HSQC) spectra for representative samples were acquired. For HSQC data, a relaxation delay equal to 1.5s was used between acquisitions and a refocusing delay of 3.45ms was implemented. In general, 3584 data points in the direct dimension and 360 data points in the indirect dimension with 144 scans per increment were acquired with spectral widths of 26 ppm in F2 and 83 ppm in F1 (^13^C). All NMR data were processed using Topspin 3.1 software (Bruker Analytik, Rheinstetten, Germany). All FIDs were subjected to an exponential line-broadening of 0.3 Hz. Upon Fourier transformation, each spectrum was manually phased, baseline corrected, and referenced to the internal standard TMSP at 0.0 ppm.

### Statistical analysis

Principal components analysis (PCA) was performed to determine the reproducibility of the extraction and sample stability and to look for metabolic differences in the Sumatran rhinoceros population pre- and post-disease onset. From ^1^H NMR spectra, spectral bin intensities tables were generated using AMIX (version 3.9.11; Bruker Biospin Inc., Billerica, MA). The spectra from 0.5 to 10.0 ppm, excluding the region of the residual water resonance (4.7–4.9 ppm) and contaminants identified in the blanks, methanol (3.35–3.36 ppm) and chloroform (7.67–7.69 ppm), were reduced into 980 uniform bins in 0.01 ppm wide. Signal intensities were summed for integration, and the spectra were normalized to constant total intensity of the spectral area. PCA analyses were performed using the spectral bin intensities tables (S1 File). The scores from each PCA analysis were exported to Microsoft Excel and differences between groups were based on PC1 and PC2 scores were assessed using Student t-tests. Though data is not presented, the autocorrelation and partial autocorrelation function plots were performed on PC1 and PC2 scores using JMP12 software (SAS Institute Inc., Cary, NC).

The spectral bin intensities tables were further analyzed using a univariate approach, based on bin-by-bin differences between healthy and unhealthy animals’ spectra. In order to identify the NMR peaks that are significantly different between healthy and unhealthy samples, ^1^H significant difference spectra (SDS) were generated [16, 17]. Pairwise differences within each bin were compared using Student’s t-test. The false discovery rate (FDR) was controlled at 0.05 level using the Benjamini Hochberg method [27]. The SDS plot was generated by taking the mean difference between the two groups, healthy vs. unhealthy. Metabolites were assigned to those significant bins based on the chemical shifts as described below.

### Metabolite assignments

Metabolites found in serum polar extracts were assigned based on one-dimensional (1D) ^1^H and two-dimensional (2D) ^1^H-^13^C NMR experiments. Peaks were assigned by comparing the chemical shifts and spin-spin couplings with reference spectra found in databases, such as the Human Metabolome Database (HMDB)[28], the Madison metabolomics consortium database (MMCD)[29], the biological magnetic resonance data bank (BMRB)[30], and Chenomx® NMR Suite profiling software (Chenomx Inc. version 8.1).

### Serum ferritin assay

Serum samples from Rhino-1 (n = 28) and Rhino-2 (n = 16) collected over a period of 8 and 7 years, respectively, were analyzed for ferritin concentrations by Kansas State University. The enzyme immunoassay employs an equine anti-ferritin antibody (S-6146, Sigma Aldrich) and black rhino ferritin as a standard and was previously validated for measuring circulating concentrations of ferritin in black rhinos [31].

## Results and Discussion

### Metabolic differences between healthy and unhealthy Sumatran rhinoceroses

**Fig 1:**
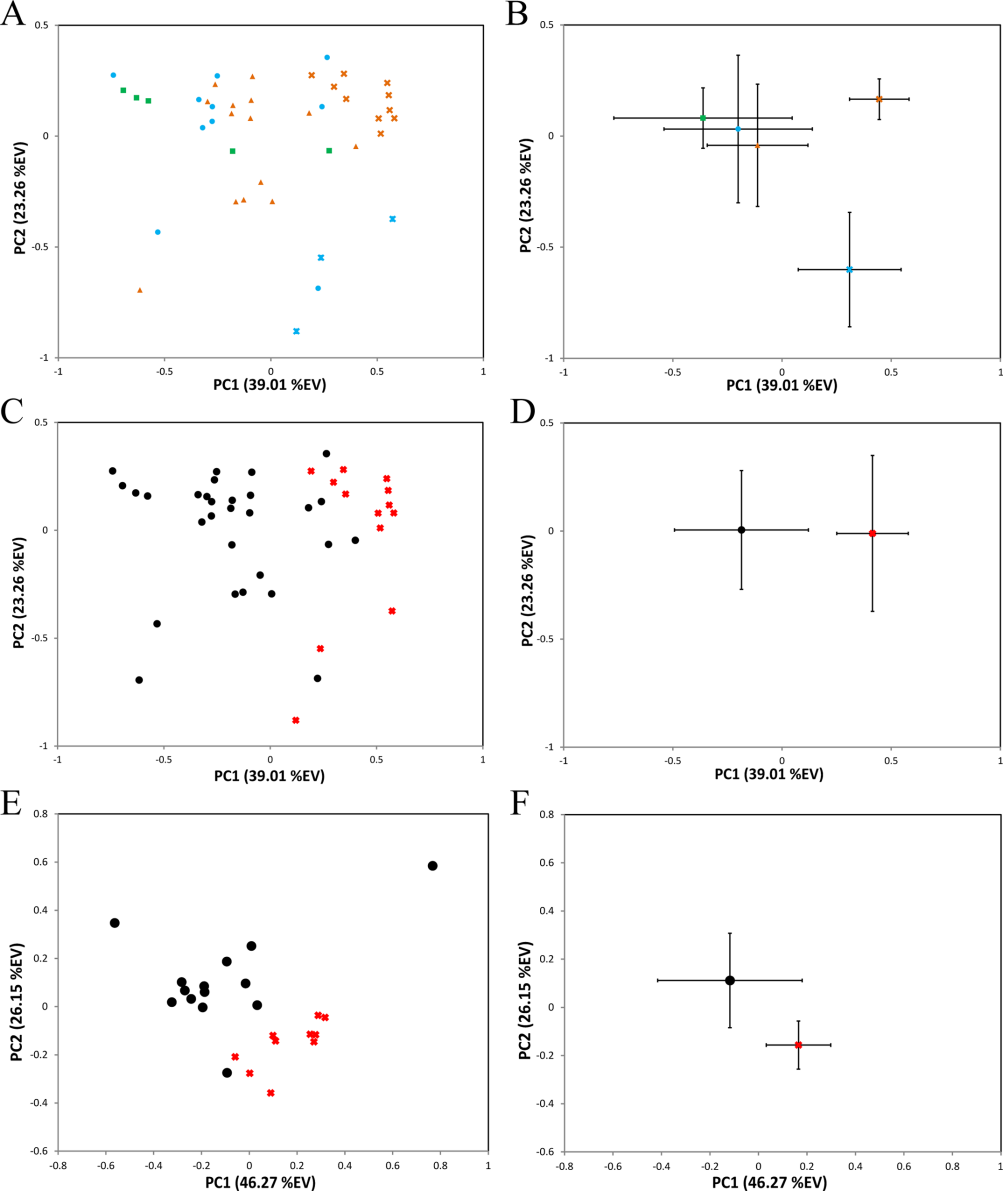
Multivariate PCA analysis of serum metabolome from Sumatran rhinoceroses. (A) PCA scores plots indicating the relationship between three rhinoceroses: Rhino-1 (red), Rhino-2 (blue), Rhino-3 (green), and their health status (x-healthy, ●-unhealthy). (B) The average PC scores of each animal corresponding to the plot (A). The error bars are indicating the standard deviations. (C) PCA scores plot (A) based on the health status of animals (x-Healthy-black, ●-Unhealthy). (D) The average scores of each animal corresponding to the plot (C). (E) PCA scores plots indicating the Rhino-1 health status (x-healthy, ●-unhealthy), and the average PC scores (F).

To our knowledge this study is the first attempt to characterize metabolomic changes associated with hemochromatosis in any wildlife species. Comparisons of metabolic profiles among three zoo animals were assessed in PCA scores plot (Fig 1A and 1B). The first component (PC1) in PCA scores plot separated the healthy samples from unhealthy samples from Rhino-1 (PC1: *p* = 8.73x10^-7^) and Rhino-2 (PC1: *p* = 3.59x10^-2^) (Fig 1B). The separation in PC2 direction between the unhealthy animals is most likely caused by individual differences with regard to factors like the progression of disease, age, and development of secondary problems associated with hemochromatosis progression (*e*.*g*. compromised liver and/or renal function and overall body wasting).

The clustering of the two study groups, healthy and unhealthy, became more apparent upon the removal of the identity of the animals (Fig 1C and 1D). The separation in PC1 direction seems to indicate the presence of metabolic differences between all the healthy and unhealthy samples, however, other potential factors including individual animal’s differences unbalanced number of samples in each group, *etc*. may influence this result. For this reason, further analysis was performed on samples only from Rhino-1, which had the largest number of samples from one animal. In the PCA analysis of Rhino-1 samples, a clear grouping was observed based on the health status (Fig 1E and 1F), no time correlation based on the progression of the disease was observed in these samples (data not shown). The separation between the healthy and unhealthy samples were significant based on the Student’s t-test in both PC1 (*p* = 5.40x10^-3^) and PC2 (*p* = 2.77x10^-4^).

### Metabolic changes during the disease development and progression

**Fig 2:**
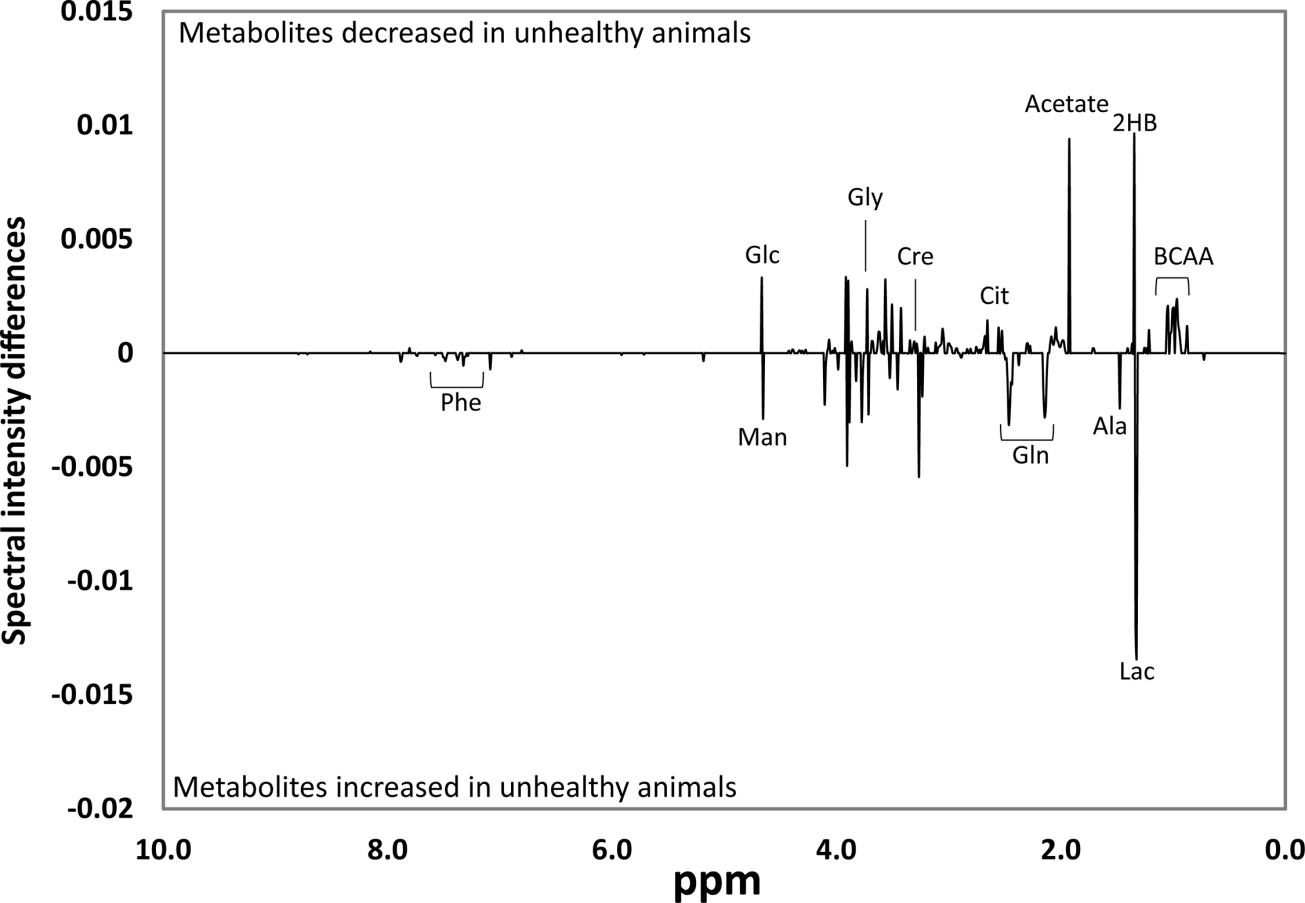
Univariate significant difference spectra (SDS) analysis. SDS spectra obtained by subtracting the mean buckets (n = 980) of the unhealthy from healthy samples. Only the buckets (n = 178) with significant alterations based on t-test with FDR correction are plotted. BCAA: branched chain amino acids, 2HB: 2-hydroxyisobutyrate, Lac: lactate, Ala: alanine, Gln: glutamine, Cit: citrate, Cre: creatine, Gly: glycine, Glc: glucose, Man: mannose, Phe: phenylalanine.

The univariate analysis of significant difference spectra (SDS) revealed 178 bins out of 980 bins with statistically significant differences between healthy and unhealthy groups (Fig 2). Significant metabolites were assigned based on these SDS bins and 1D and 2D NMR spectral annotations. A total of 18 metabolites differed in relative intensities between healthy and unhealthy animal samples (Table 2).

**Table 2:** List of metabolites that were altered significantly in unhealthy compared to healthy rhino serum samples after FDR correction.

Metabolites^a^	Bin^b^	*P* value^c^	Changes^d^	
	(ppm)	(FDR Corr)		Function
Ile	1.015	1.02E-11	↓	Essential amino acid, BCAA, protein catabolism
Val	1.055	8.10E-10	↓	Essential amino acid, BCAA, protein catabolism
Gly	3.565	2.59E-03	↓	Amino acid metabolism
Leu	0.955	1.04E-05	↓	Essential amino acid, BCAA, protein catabolism
Gln	2.145	3.46E-05	↑	Amino acid metabolism
Asn	2.885	1.74E-03	↓	Amino acid metabolism
Ala	1.475	9.02E-05	↑	Amino acid metabolism
Phe	7.325	9.61E-06	↑	Essential amino acid, AAA,
Mannose	5.185	9.57E-07	↑	Carbohydrate sugar, Glycoprotein
Glucose	5.235	1.55E-03	↓	Carbohydrate sugar
Creatine	3.045	5.21E-03	↓	Creatinine synthesis in liver
Phosphocreatine	3.055	1.00E-07	↓	Creatinine synthesis in liver
Creatinine	4.065	1.81E-06	↓	Creatinine synthesis in liver
Pyruvate	2.375	2.20E-05	↑	Krebs cycle
Citrate	2.525	2.41E-08	↓	Krebs cycle, Fatty acid synthesis
Lactate	4.105	5.25E-07	↑	Krebs cycle
2-Hydroxyisobutyrate	1.365	8.61E-08	↓	
Acetate	1.925	1.71E-06	↓	

^a^ Ile: isoleucine, Val: valine, Gly: glycine, Leu; leucine, Gln: glutamine, Asn: asparagine, Ala: alanine, Phe: phenylalanine.

^b^ The chemical shift of the bin used for the p value calculation.

^c^ The FDR corrected alpha values.

^d^ Increased (↑) and decreased (↓)in unhealthy samples compared to healthy samples.

**Fig 3:**
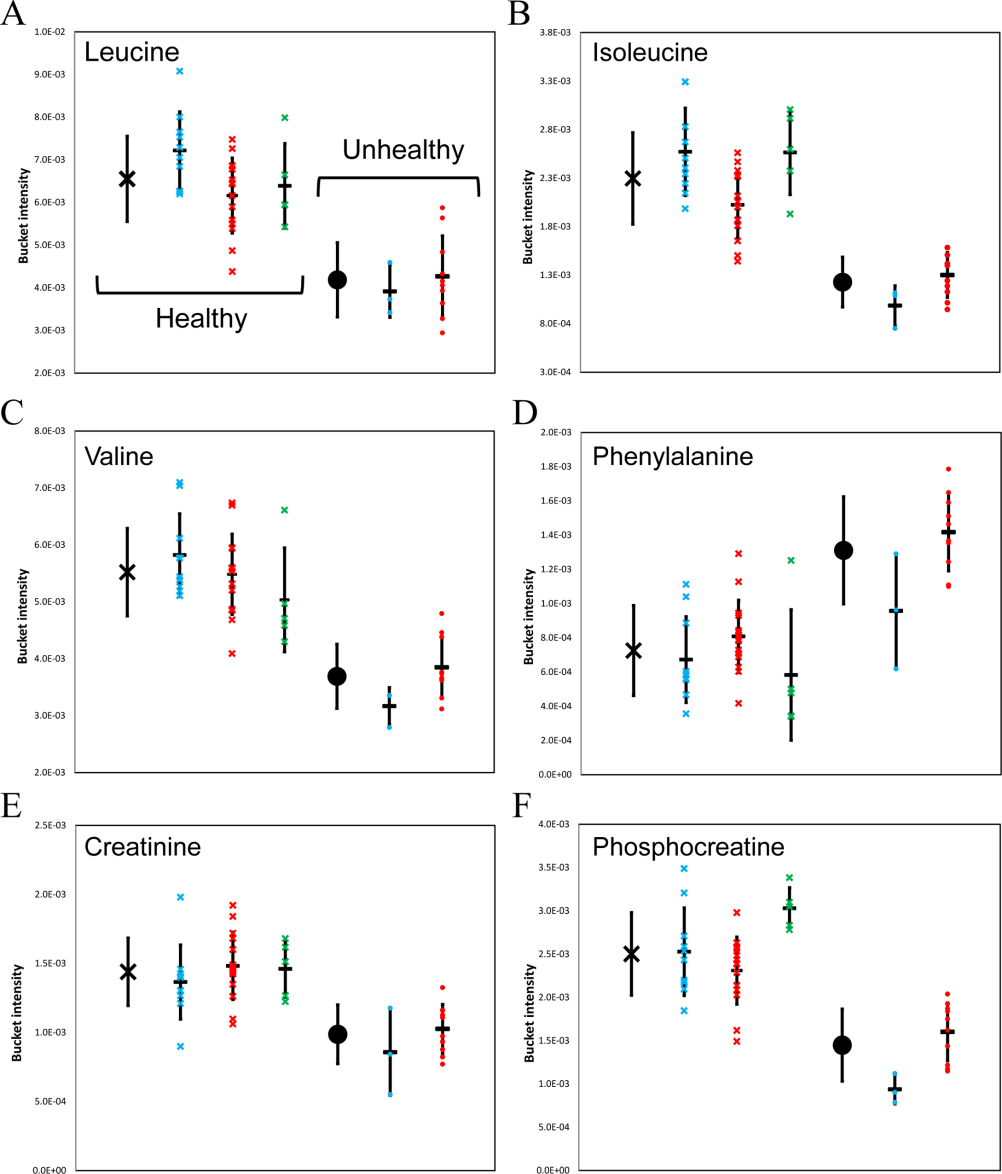
Metabolite changes in relation to health status of rhinoceroses. Bucket intensities of each altered metabolite from three rhinoceroses: Rhino-1 (red), Rhino-2 (blue), Rhino-3 (green), and their health status (x-healthy, ●-unhealthy). The averages of all healthy or unhealthy samples are shown in black. (A) leucine, (B) isoleucine, (C) valine, (D) phenylalanine, (E) creatinine, (F) phosphocreatine.

Significant decreases in branched-chain amino acids ((BCAAs) leucine, isoleucine, and valine) were observed in unhealthy samples from Rhino-1 and -2 compared to healthy samples (Fig 3A–3C). The high clearance rate of plasma BCAAs in humans with liver cirrhosis has been identified previously[32]. A number of studies have demonstrated the benefits of BCAA supplementations in albumin synthesis[33], nutritional status[34], glucose metabolism[35, 36], and decreasing the frequency of hepatocellular carcinoma [37]. More recently, the oral supplementation of BCAA has been shown to reduce hepatic iron accumulation and oxidative stress in mice [38].

In addition to the decrease in BCAA, the increase in circulating aromatic amino acids (AAA) has been reported in several cases of hepatic failure [39–42]. The decrease in Fischer-ratio (BCAA/AAA ratio) is suggested to be due to the induction of muscle catabolism and reduction in AAA breakdown in the dysfunctional liver [43]. The observation of increased phenylalanine levels (Fig 3D) along with the rapid clearance of BCAA in both the Rhino-1 and -2 unhealthy samples strongly correlate with these previous observations in liver failure associated with hepatic encephalopathy.

Significant decreases in creatine, creatinine (Fig 3E), and phosphocreatine (Fig 3F) were identified in unhealthy samples (Table 1). Though there are limitations in the use of serum creatinine concentrations as a tool for monitoring liver disease[44], it has been incorporated into the panel of tests employed to monitor the decline of hepatic function in humans [45–47]. Based on these results that indicate reduced creatine, phosphocreatine and creatinine concentrations along with altered BCAA and AAA in serum collected from rhinoceroses in liver failure, it may be prudent to include tests for these metabolites when monitoring rhinoceroses with suspected liver problems.

Reproducibility across multiple aspects of the study is a key to both the overall success of the study and also to the ability to interpret the results obtained. To assess the reproducibility of the sample extraction, sample stability and analytical methods, quality control samples were prepared and analyzed along with the test samples. The PCA analysis of all the samples in this study indicated the high repeatability of the sample processing and analytical methods (S1 Fig). The spectral median relative standard deviation[48] of PCM was determined to be 13.02%.

As the metabolic signature of the Sumatran Rhino had not previously been studied, we not only investigated the metabolic differences between the healthy and unhealthy samples, but also attempted to gain insight into the most abundant metabolites present in rhinoceros serum. A total of 34 metabolites were assigned based on the 1D and 2D HSQC NMR experiments (S2 Fig) and the representative spectra demonstrates the complexity that arises from the untargeted approach at investigating the polar serum metabolites.

In addition to understanding the metabolic differences associated with the diseased state, we were also interested in investigating the degree of separation that might occur between the metabolomes of rhinoceroses in different environments with different diets knowing that such variables can be significant confounding factors in metabolomics studies. Assessments of metabolic differences due to the living environment were performed by comparing healthy samples from the Sumatran rhinoceroses at the Cincinnati Zoo and those from the rhinoceroses at the SRCC (S3 Fig). The grouping of three zoo animals (Rhino-1 through -3) suggested that the effects from difference in ages of animal, time of collections (season/ year), or in diet within the zoo on serum metabolome are minimal. The largest separation was found in PC1 (*p* = 7.67x10^-5^) between the animals in the SRCC (Rhino-4 through -7) and the zoo (Rhino-1 through -3), indicating the different countries had the most profound impact on metabolomic differences, most likely due to very different browse diets being fed.

Finally, the sensitivity of the NMR-based metabolomics assay was compared to the currently available assays. The serum ferritin concentrations of Rhino-1 and Rhino-2 were evaluated over time (S4 Fig). Though the overall trends show increased levels of ferritin in both animals following disease diagnosis, significant fluctuations were also observed during period that the animals were healthy. These observations emphasize the difficulty in diagnosing ISD in the rhinoceros species by the traditional methods, and highlight the need for additional diagnostic assays that can be used both in combination with serum ferritin monitoring or as stand-alone surveillance techniques

## Conclusion

The measurement of serum ferritin concentrations via ELISA currently is the primary method employed for monitoring total body iron load and the progression of ISD in the rhinoceros. Although serum ferritin does increase in Sumatran rhinoceroses suffering from hemochromatosis, its value as a diagnostic biomarker of this disease in this species has recently been questioned [8]. Therefore, the identification of other biomarkers that could provide a more definitive diagnosis would be of great value for the rhinoceros and other wildlife species susceptible to this disease. Data generated in this study are encouraging and suggest that metabolomics can play an important role in helping to identify potential biomarkers of disease progression and/or organ dysfunction in wildlife. Ferritin is one of the main intercellular iron storage proteins and the increased level of ferritin has been observed during the ISD development in multiple species including rhinoceros. Ferritin level is regulated through porphyrin metabolism pathway which includes heme protein synthesis. As both glycine and BCAA are involved in this pathway, the significant decrease in this combination of amino acids may also be due to the alteration in ferritin production. The induction of ferritin production due to the high level of free-iron, could be utilizing all the free glycine and BCAA in the pool. The primary cause of these alterations in the serum metabolome of unhealthy rhinoceroses remains unknown.

In order to pinpoint the cause of ISD, additional studies that include both larger sample sizes and a more diverse sampling from multiple animals is needed. In spite of the noted limitations, this study has successfully demonstrated the capability of NMR-based metabolomics to detect changes in the health of a non-model species, the Sumatran rhinoceros. Alterations of 18 serum metabolites, many of which have previously been linked to liver dysfunction in other species, were observed in the unhealthy animals. The specificity of the observed metabolic changes to the development of ISD will be investigated with a study consisting of a larger population size. Although the declining rhinoceros species population makes increasing the sample size challenging, it also highlights the importance of this type of study in helping to ultimately reduce the number of rhinoceros deaths. Nonetheless, the results here indicate that the serial monitoring of the blood metabolome over an extended period of time would allow each animal to serve as its own control to evaluate health status. This time-series analysis approach for monitoring metabolic changes during disease progression may lead to the development of new diagnostic methods in non-model species.

## Supporting Information

S1 Fig Quality control assessment. PCA score plot of test samples and quality control (QC) samples used in this study. Identical pooled plasma samples (PCM) were extracted along with the study samples in each batch. Total of 5 PCM (+) and 47 test samples: Rhino-1(x, red), Rhino-2(●, blue), Rhino-3(∆, green), Rhino-4~7(♦, yellow), were extracted in this study. The PCA scores plot and the calculated relative standard deviation (RSD) of the PCM (13.02%) indicated a high degree of reproducibility between the extraction batches
(PDF)

S2 Fig Serum metabolome of Sumatran rhinoceros (*Dicerorhinus sumatrensis*). A representative ^1^H NMR spectrum of serum polar extract from Sumatran rhinoceros and metabolite assignments that have been confirmed with 2D HSQC NMR experiments in two sections A) 0.5–4.6ppm, and B) 5.0–8.6ppm. Assignments: 1) 2-hydroxyvalerate, isoleucine, leucine, valine, 2) isobutyrate, 3) propylene glycol, 4) 3-hydroxybutyrate, 5) 3-hydroxyisovalerate, 6) lactate, 7) 2-hydroxyisobutyrate, 8) alanine, 9) suberate, 10) lysine, 11) acetate, 12) glutamate, 13) succinate, pyruvate, 14) glutamine, 15) citrate, 16) asparagine, 17) creatine, phosphocreatine, 18) histidine, 19) betaine, 20) glycine, 21) threonine, 22) mannose, 23) glucose, 24) tyrosine, 25) phenylalanine, 26) hippurate, 27) tryptophan, 28) benzoate, 29) formate.
(PDF)

S3 Fig Environmental effect in Sumatran rhinoceros serum metabolome. PCA scores plot (A) and the PCA score average (B) of serum samples from the animals maintained at the Cincinnati Zoo, USA, Rhino-1(x), Rhino-2(●), Rhino-3(∆-green), and the Sumatran Rhino Conservation Center, Malaysia, Rhino-4~7(♦-yellow) indicating the metabolic differences associated with the different living environment.
(PDF)

S4 Fig The serum ferritin concentrations from captive rhinoceroses. The changes in serum ferritin concentrations s from Rhino-1 (♦) and Rhino-2 (●) are plotted over time. The disease diagnostic time point is indicated by the (*) for both animals and the health status is indicated in black (healthy) and red (sick).Though the sampling time period over laps with that for samples analyzed in this study, the samples were not all identical matches due to the limited amount of samples available from any given date.
(PDF)

S1 File The spectral bin table of all the Sumatran rhinoceroses serum samples used in this study. (ZIP)
