# Supplementary material for: Feasibility Study of NMR Based Serum Metabolomic Profiling to Animal Health Monitoring: A Case Study on Iron Storage Disease in Captive Sumatran Rhinoceros (Dicerorhinus sumatrensis)
Source: PLoS One. 2016 May 27;11(5):e0156318. doi: 10.1371/journal.pone.0156318 (PMC4883739; doi:10.1371/journal.pone.0156318)

Figure S1. Quality control assessment. PCA score plot of test samples and quality control (QC) samples used in this study. Identical pooled plasma samples (PCM) were extracted along with the study samples in each batch. Total of 5 PCM (+) and 47 test samples: Rhino-1(x, red), Rhino-2(●, blue), Rhino-3(Δ, green), Rhino-4~7(♦, yellow), were extracted in this study. The PCA scores plot and the calculated relative standard deviation (RSD) of the PCM (13.02%) indicated a high degree of reproducibility between the extraction batches

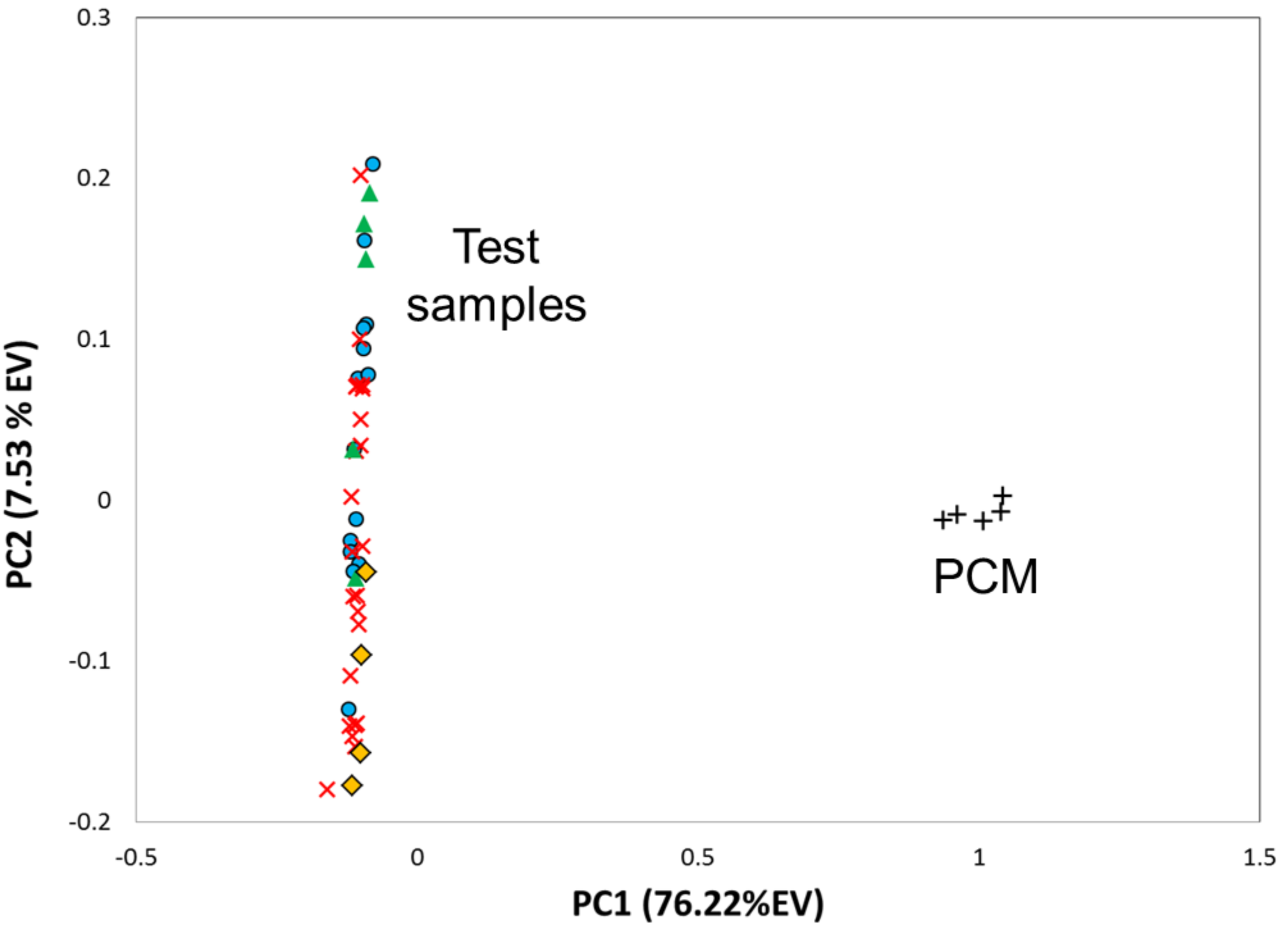

Supplement: S1 Fig — PCA score plot of test samples and quality control (QC) samples used in this study. Identical pooled plasma samples (PCM) were extracted along with the study samples in each batch. Total of 5 PCM (+) and 47 test samples: Rhino-1(x, red), Rhino-2(●, blue), Rhino-3(∆, green), Rhino-4~7(♦, yellow), were extracted in this study. The PCA scores plot and the calculated relative standard deviation (RSD) of the PCM (13.02%) indicated a high degree of reproducibility between the extraction batches (PDF) [file pone.0156318.s001.pdf]
