# Supplementary material for: Feasibility Study of NMR Based Serum Metabolomic Profiling to Animal Health Monitoring: A Case Study on Iron Storage Disease in Captive Sumatran Rhinoceros (Dicerorhinus sumatrensis)
Source: PLoS One. 2016 May 27;11(5):e0156318. doi: 10.1371/journal.pone.0156318 (PMC4883739; doi:10.1371/journal.pone.0156318)

Figure S2. Serum metabolome of Sumatran rhinoceros (*Dicerorhinus sumatrensis*). A representative ^1H NMR spectrum of serum polar extract from Sumatran rhinoceros and metabolite assignments that have been confirmed with 2D HSQC NMR experiments in two sections A) 0.5-4.6ppm, and B) 5.0-8.6ppm. Assignments: 1) 2-hydroxyvalerate, isoleucine, leucine, valine, 2) isobutyrate, 3) propylene glycol, 4) 3-hydroxybutyrate, 5) 3-hydroxyisovalerate, 6) lactate, 7) 2-hydroxyisobutyrate, 8) alanine, 9) suberate, 10) lysine, 11) acetate, 12) glutamate, 13) succinate, pyruvate, 14) glutamine, 15) citrate, 16) asparagine, 17) creatine, phosphocreatine, 18) histidine, 19) betaine, 20) glycine, 21) threonine, 22) mannose, 23) glucose, 24) tyrosine, 25) phenylalanine, 26) hippurate, 27) tryptophan, 28) benzoate, 29) formate.

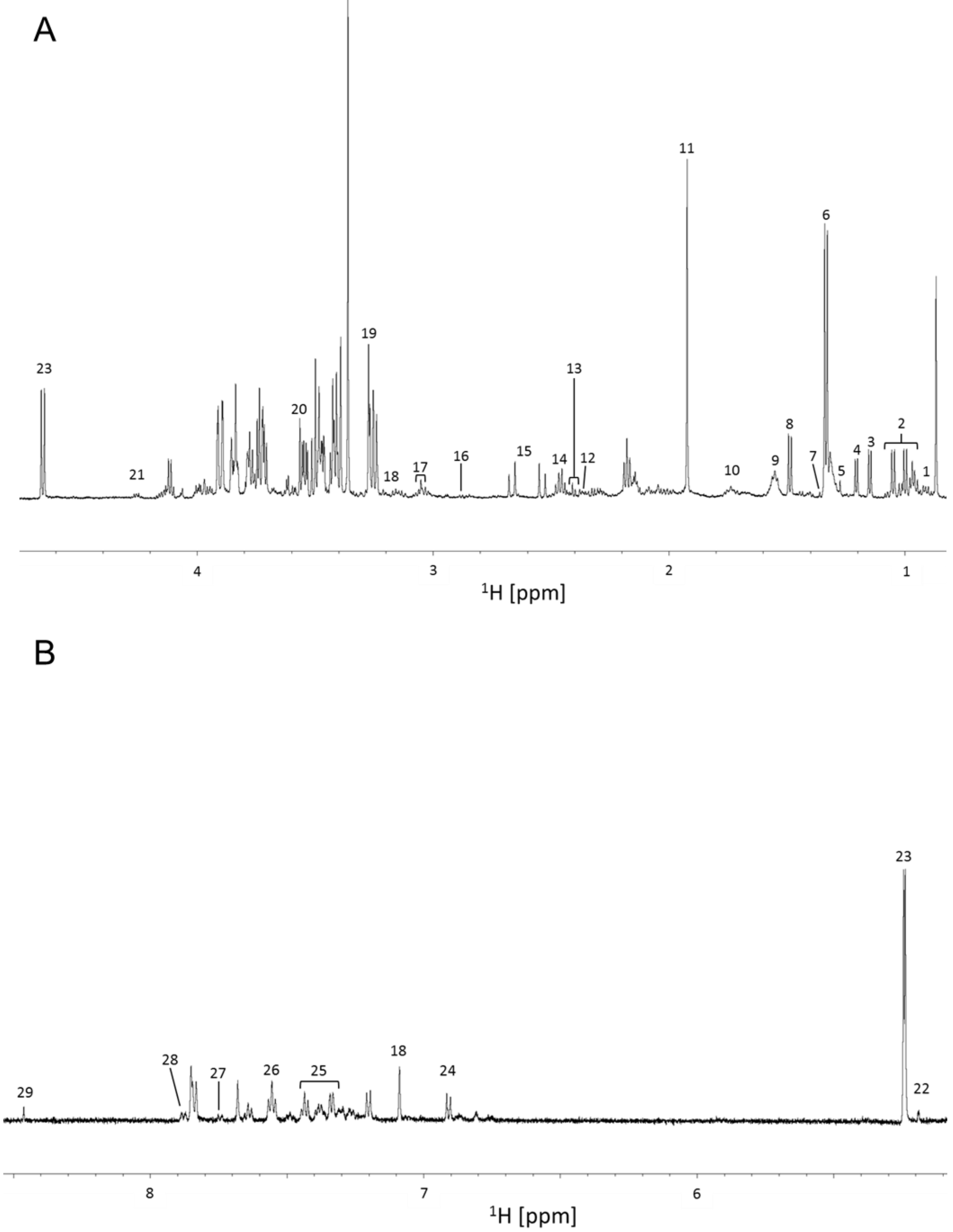

Supplement: S2 Fig — A representative 1H NMR spectrum of serum polar extract from Sumatran rhinoceros and metabolite assignments that have been confirmed with 2D HSQC NMR experiments in two sections A) 0.5–4.6ppm, and B) 5.0–8.6ppm. Assignments: 1) 2-hydroxyvalerate, isoleucine, leucine, valine, 2) isobutyrate, 3) propylene glycol, 4) 3-hydroxybutyrate, 5) 3-hydroxyisovalerate, 6) lactate, 7) 2-hydroxyisobutyrate, 8) alanine, 9) suberate, 10) lysine, 11) acetate, 12) glutamate, 13) succinate, pyruvate, 14) glutamine, 15) citrate, 16) asparagine, 17) creatine, phosphocreatine, 18) histidine, 19) betaine, 20) glycine, 21) threonine, 22) mannose, 23) glucose, 24) tyrosine, 25) phenylalanine, 26) hippurate, 27) tryptophan, 28) benzoate, 29) formate. (PDF) [file pone.0156318.s002.pdf]
