# Supplementary material for: Feasibility Study of NMR Based Serum Metabolomic Profiling to Animal Health Monitoring: A Case Study on Iron Storage Disease in Captive Sumatran Rhinoceros (Dicerorhinus sumatrensis)
Source: PLoS One. 2016 May 27;11(5):e0156318. doi: 10.1371/journal.pone.0156318 (PMC4883739; doi:10.1371/journal.pone.0156318)

Figure S3. Environmental effect in Sumatran rhinoceros serum metabolome. PCA scores plot (A) and the PCA score average (B) of serum samples from the animals maintained at the Cincinnati Zoo, USA, Rhino-1(x), Rhino-2(●), Rhino-3(Δ-green), and the Sumatran Rhino Conservation Center, Malaysia, Rhino-4~7(♦-yellow) indicating the metabolic differences associated with the different living environment.

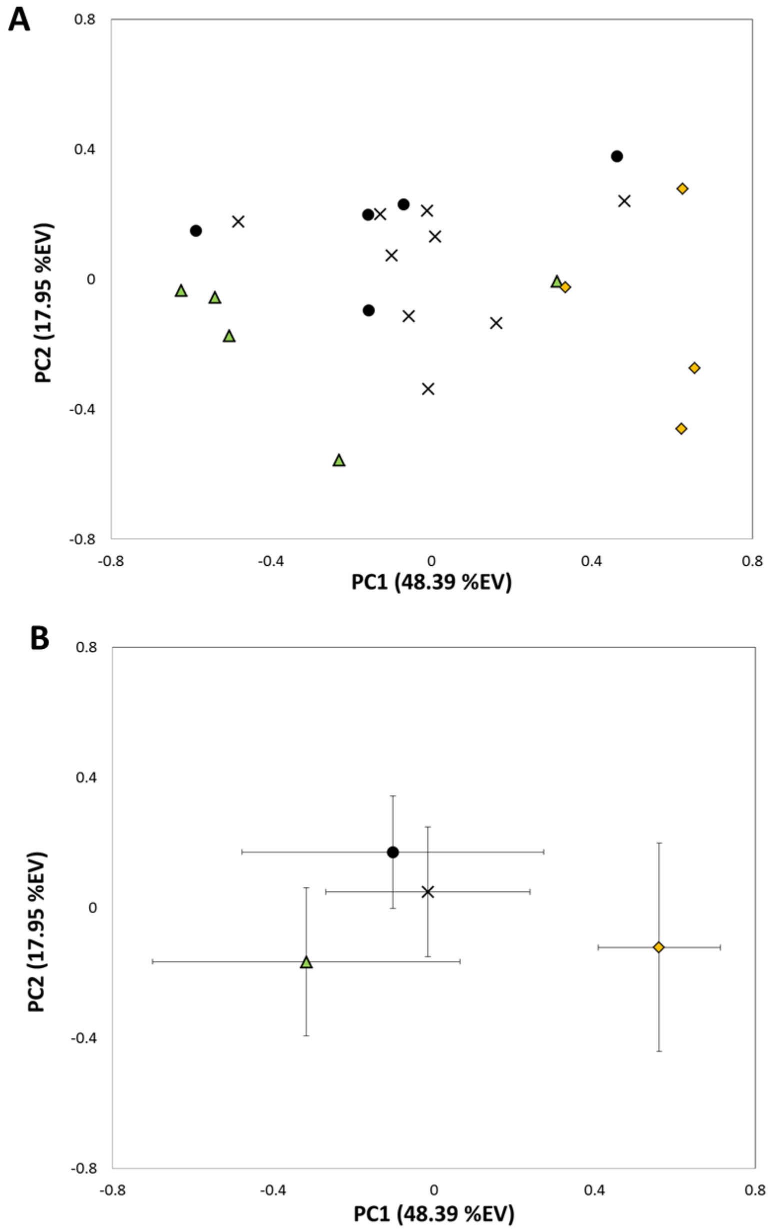

Supplement: S3 Fig — PCA scores plot (A) and the PCA score average (B) of serum samples from the animals maintained at the Cincinnati Zoo, USA, Rhino-1(x), Rhino-2(●), Rhino-3(∆-green), and the Sumatran Rhino Conservation Center, Malaysia, Rhino-4~7(♦-yellow) indicating the metabolic differences associated with the different living environment. (PDF) [file pone.0156318.s003.pdf]
