# Supplementary material for: Feasibility Study of NMR Based Serum Metabolomic Profiling to Animal Health Monitoring: A Case Study on Iron Storage Disease in Captive Sumatran Rhinoceros (Dicerorhinus sumatrensis)
Source: PLoS One. 2016 May 27;11(5):e0156318. doi: 10.1371/journal.pone.0156318 (PMC4883739; doi:10.1371/journal.pone.0156318)

S4 Fig. The serum ferritin concentrations from captive rhinoceroses. The changes in serum ferritin concentrations s from Rhino-1 (♦) and Rhino-2 (●) are plotted over time. The disease diagnostic time point is indicated by the (*) for both animals and the health status is indicated in black (healthy) and red (sick).Though the sampling time period over laps with that for samples analyzed in this study, the samples were not all identical matches due to the limited amount of samples available from any given date.

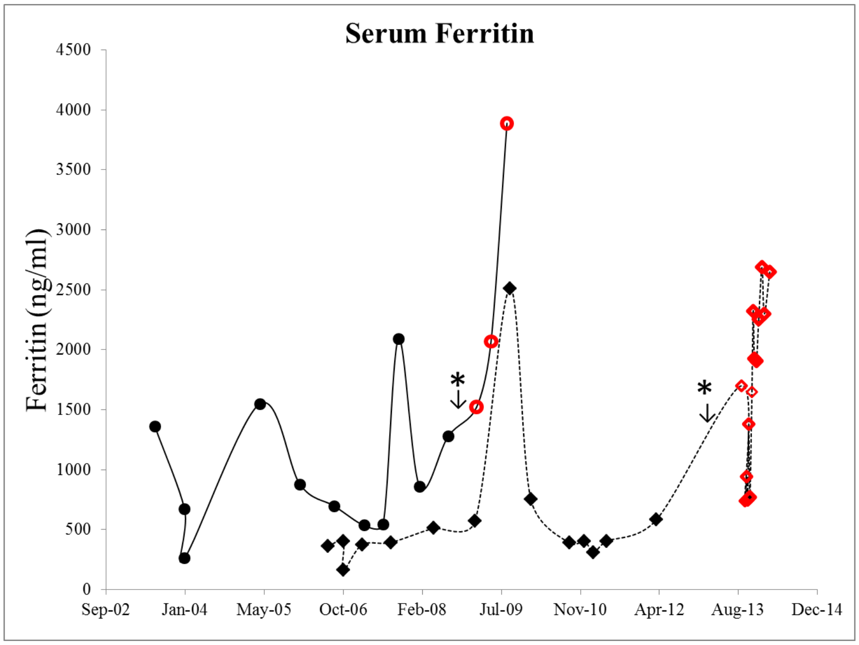

Supplement: S4 Fig — The changes in serum ferritin concentrations s from Rhino-1 (♦) and Rhino-2 (●) are plotted over time. The disease diagnostic time point is indicated by the (*) for both animals and the health status is indicated in black (healthy) and red (sick).Though the sampling time period over laps with that for samples analyzed in this study, the samples were not all identical matches due to the limited amount of samples available from any given date. (PDF) [file pone.0156318.s004.pdf]
